# Molecular Heterogeneity and Immunosuppressive Microenvironment in Glioblastoma

**DOI:** 10.3389/fimmu.2020.01402

**Published:** 2020-07-17

**Authors:** Syreeta DeCordova, Abhishek Shastri, Anthony G. Tsolaki, Hadida Yasmin, Lukas Klein, Shiv K. Singh, Uday Kishore

**Affiliations:** ^1^Biosciences, College of Health and Life Sciences, Brunel University London, London, United Kingdom; ^2^Central and North West London NHS Foundation Trust, London, United Kingdom; ^3^Immunology and Cell Biology Laboratory, Department of Zoology, Cooch Behar Panchanan Barma University, Cooch Behar, India; ^4^Department of Gastroenterology and Gastroenterology Oncology, University Medical Centre, Göttingen, Germany

**Keywords:** glioblastoma, microenvironment, brain tumor, immunity, microglia, astrocytes

## Abstract

Glioblastoma (GBM) is the most aggressive primary brain tumor in adults, with a poor prognosis, despite surgical resection combined with radio- and chemotherapy. The major clinical obstacles contributing to poor GBM prognosis are late diagnosis, diffuse infiltration, pseudo-palisading necrosis, microvascular proliferation, and resistance to conventional therapy. These challenges are further compounded by extensive inter- and intra-tumor heterogeneity and the dynamic plasticity of GBM cells. The complex heterogeneous nature of GBM cells is facilitated by the local inflammatory tumor microenvironment, which mostly induces tumor aggressiveness and drug resistance. An immunosuppressive tumor microenvironment of GBM provides multiple pathways for tumor immune evasion. Infiltrating immune cells, mostly tumor-associated macrophages, comprise much of the non-neoplastic population in GBM. Further understanding of the immune microenvironment of GBM is essential to make advances in the development of immunotherapeutics. Recently, whole-genome sequencing, epigenomics and transcriptional profiling have significantly helped improve the prognostic and therapeutic outcomes of GBM patients. Here, we discuss recent genomic advances, the role of innate and adaptive immune mechanisms, and the presence of an established immunosuppressive GBM microenvironment that suppresses and/or prevents the anti-tumor host response.

## Introduction

Glioblastoma (GBM) is the most common primary brain tumor with an annual incidence of 3.19 per 100,000 population (1). GBM is a Grade IV astrocytoma, characterized by uncontrolled cellular proliferation, local infiltration, extensive genomic instability, tendency for necrosis, angiogenesis, and resistance to therapy. Histopathologically, GBM is composed of a heterogeneous cell population, consisting of differentiated and undifferentiated tumor cells, along with differences in morphology and capacity for self-renewal and proliferation (2, 3). Despite aggressive treatment including surgical resection and radiotherapy with concomitant chemotherapy, prognosis remains poor due to GBM recurrence, with a median survival of 14.6 months (4). In molecular terms, this poor prognosis is mostly characterized by dysregulation of many key signaling pathways involving cell survival, growth, proliferation and apoptosis due to genomic mutations (5). GBM is a robust malignant tumor, distinguished by its local invasion pattern (6, 7). Generally, GBM does not metastasize extracranially; however, there have been rare cases in which 0.44% of GBM have spread to other parts of the body usually when patients have undergone craniotomy (8, 9).

GBM is highly invasive, lack clear margins, and therefore, poses a challenge for complete surgical resection and almost inevitably recurs in patients who have been treated. Despite recent advances in genomics, chemotherapy, immunotherapy, and technological approaches to cancer models, the treatment outcome for GBM patients has remained consistently poor. Clinical symptoms vary and depend on size and location of tumor; it may include headache, nausea, dizziness, confusion, speech difficulties, and change in personality, new onset of seizures and focal neurological deficit. The tumor is generally located in the frontal and temporal lobes of the brain and can also rarely occur in the brainstem, cerebellum and spinal cord (10, 11). GBM is most often *de novo* i.e., primary GBM, which account for ~90% of GBM cases and are predominately found in patients older than 45 years (5). The remaining 10% of GBM cases develop from a lower-grade tumor progressing to a higher-grade malignancy (secondary GBM) over a 5–10 year period, and is primarily present in patients younger than 45 years. These subtypes have distinct genetic aberrations but are histologically indistinguishable (5, 12, 13).

Despite advances in our understanding of cancer biology, managing GBM remains a challenge. It is important to understand why treatment for GBM is largely ineffective; it is mainly due to the heterogeneous nature of the tumor microenvironment. It has not been possible to produce appropriate cancer models for GBM that would help us study the properties by which GBM is promoted and sustained. Therefore, it is vital to study the role of the immune system in the GBM microenvironment. This review aims to analyze the recent genomic advances in dissecting the considerable molecular and cellular heterogeneity in GBM and the innate and adaptive immune mechanisms that are suppressed, which ultimately contribute to tumorigenesis.

## Genomic Landscape of the GBM Microenvironment

GBM shows considerable cellular and molecular heterogeneity, both between patients and within the tumor microenvironment itself. GBM subtyping via histological examinations is a poor prognostic indicator for gliomas. Glioma is an overarching term used for brain tumors of glial cells: astrocytes, glioblastoma, oligodendrocytes, oligodendroglioma, ependymal cells, ependymoma, and was improved by combining histology with molecular genotyping of key markers (e.g., iso-citrate dehydrogenase (IDH), ATP-dependent helicase (ATRX), Lys-27-Met mutations in histone 3 (H3K27M), p53 mutations, and 1p/19q chromosomal deletion (14). However, the era of genomics and next generation sequencing (NGS) has led to a greater understanding of the formation and pathogenesis of these tumors by identifying core molecular pathways affected, facilitating the design of novel treatment regimens. The Cancer Genome Atlas (TCGA) network was among the first to conduct a major genomic study interrogating 33 different types, with particular emphasis on GBM, leading to the whole genome characterization and molecular genotyping of 600 GBM and 516 other low-grade gliomas (15). Novel genomic variations were identified, e.g., deletions of neurofibromin gene (NF1) and parkin RBR E3 ubiquitin protein ligase (PARK2) as well as copy number variations (CNVs) of AKT serine/threonine kinase 3 (AKT3) and other single nucleotide variations (SNVs). Furthermore, patients who had undergone treatment were shown to have higher genetic variability in their recurrent tumors than untreated patients, showing additional layers of complexity in the pathogenesis and progression of GBM. These data allowed the TCGA to group GBM into distinct molecular subtypes (16). Subsequent studies further refined this classification using additional genomic and transcriptomic data to give the following three most clinically relevant molecular subtypes of GBM: proneural (PN), mesenchymal (MSC), and classical (CL) (Table 1). This classification was based on platelet-derived growth factor receptor A (PDGFRA) gene/IDH mutation, NF1 mutation, and epidermal growth factor receptor (EGFR) expression, respectively (15, 22). EGFR is also an important marker for proliferation and MSC subtype (23).

**Table 1:** Adult (WHO Grade IV) Glioblastoma multiforme (GBM) subtypes defined by genomic, transcriptome and epigenomic markers.

**GBM phenotype**	**Methylation status**	**Genotypic/phenotypic abnormality**	
**Proneural (PN)**	G-CIMP+^*^	IDH1/IDH2 mutations	Ch10 deletion
	MGMT gene promoter (high)	ARTX mutation	MYC
		TP53 mutation	CDKN2A/CDKN2B deletion
	G-CIMP–^*^	IDH1 wildtype	RTKI
		TERT promoter mutation PDGRFA amplification Ch7 insertion/chr10 deletion	CDK4 amplification DLL3, OLIG2 and NKX2-2
**Classic (CL)**	Cluster M3^*^ MGMT gene promoter (moderate)	EGFR amplification/mutation RTKII	CDKN2A/CDKN2B deletion PTEN deletion
		EGFRvIII	TERT promoter mutation
		Ch7 insertion/chr10 deletion	IDH1/IDH2 wildtype
**Mesenchymal (MSC)**	Cluster M1^*^	NF1 mutation	VEGRF2
		TP53 mutation	CD40, CD31, CD68
		S100A1, PTPRC TERT promoter mutation	CHI3L1/YKL-40, MET EGFR amplification (MSC subtypes)
		Ch7 insertion/chr10 deletion	**↑**NF-κB driven inflammation

*Neural “subtype” not used in classification as no gene clustering observed in several studies (15, 17–20). G-CIMP, Glioma CpG island methylator phenotype; MGMT, O^6^-methylguanine-DNA methyltransferase; TERT, Telomerase reverse transcriptase; RTKI, RTKII, Receptor tyrosine kinase I and II; EGFR, Epidermal growth factor receptor; VEGRF2, vascular endothelial growth factor receptor 2; PTPRC, Protein Tyrosine Phosphatase Receptor Type C; S100A1, S100 Calcium Binding Protein A1; MET, MET-Proto-Oncogene, Receptor Tyrosine Kinase*.

* *Methylation cluster and G-CIMP phenotype defined by Brennan et al. (21). ↑, enhanced. Ch, Chromosome. Table compiled using data from the following: Cancer Genome Atlas Research Network (16), Verhaak et al. (22), Wang et al. (15), Phillips et al. (23), Bhat et al. (24), Patel et al. (25), Noushmehr et al. (26), de Souza et al. (27), Reifenberger et al. (28), and Waker et al. (29)*.

These GBM classifications have been key in trying to associate genomic/molecular variation to clinical phenotypes, particularly in recurrent episodes and treatment failures, such as the PN-MSC subtype-switch in the tumor aggressiveness and resistance. In line with this, a recent study (where glioma cells were treated with varying concentrations of cytokines) revealed that cytokine storm in the GBM tumor microenvironment enforces PN-subtype switch to MES-subtype by transcriptional networking and induces radiation-resistance properties (24). Similarly, another study shows that post-translational modification of oncogenic transcription factors (TF) such as OLIG2, switches the proliferative nature of glioma cells into a highly invasive phenotype by controlling the inflammatory cytokine, TGF-β (30). Prognostically, GBM patients with the MSC subtype tend to have a poor survival and resistance to therapy in comparison to other subtypes. Inevitably, NF1 drives mutations and a characteristic NF-κB transcriptome profile, an important inflammatory TF that seems to be very specific to MSC subtype (17). Moreover, NF1 is an RAS-GTPase and an important tumor suppressor gene. Its disruption, through mutation or deletion, is associated with enhanced tumor aggression and invasiveness (31). Deficiency in NF1 is also key in macrophage/microglia recruitment (32–34).

Most of the early TCGA studies have utilized tissue from one single random location in the tumor, but as mentioned above, GBM has high levels of cellular heterogeneity, with several factors affecting the molecular subtype, including anatomical location. Using RNA-Seq, a single GBM sample was shown to contain cells from 3 different subtypes (25). Approximately 8% of the GBM samples contain more than one subtype. Therefore, there needs to be a refinement of these genomic approaches to characterize genetic and protein changes to both single cell and specific cell populations within the tumor (35). Understanding the nature and consequences of cellular and molecular heterogeneity in GBM is crucial in identifying new biomarkers and therapeutic interventions. To date, there has been little evidence of significant association between molecular subtype and prognosis, although recently poorer prognosis has been observed in the MSC subtype, compared to other subtypes (17). Furthermore, enhanced survival was observed in GBM samples of low heterogeneity in 20% of the total GBM samples analyzed (15).

**Figure 1:**
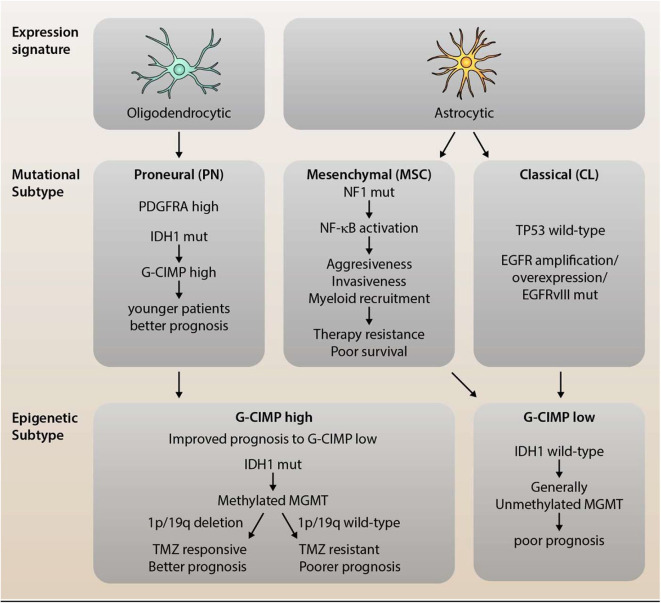
Dissection of Mutational and Epigenetic GBM Subtype Classifications. Glioblastoma (GBM) is a highly heterogeneous disease with distinct, recurring molecular subtypes that differ in their associated expression profile, mutational signature, and epigenetic modifications. GBM can be classified into three main subtypes: the proneural (PN), mesenchymal (MSC), and classical (CL) subtype. PN gliomas tend to display an expression profile resembling oligodendrocytes, high levels of PDGFRα (due to amplifications or mutations) as well as characteristic mutations in IDH1. The latter leads to an epigenetic CpG island methylator phenotype (C-GIMP), which is associated with younger patients and a better prognosis. MSC subtype tumors, on the other hand, show a high rate of NF1 mutations which, in turn, promotes NF-κB activation and, thereby, aggressiveness, invasiveness, and myeloid recruitment. This translates into a therapy resistant phenotype for MSC gliomas with poorer survival compared to the other subtypes. The third subtype is the classical subtype, which preserves wild-type p53 expression, but shows over-expression and/or mutation of EGFR. Both MSC and CL tumor cells resemble (cultured) astrocytic gene expression profiles as well as epigenetically a G-CIMP low phenotype. The distinction between G-CIMP high and low is not only prognostically relevant (as G-CIMP high shows improved prognosis), but also predictively. Methylation of MGMT, which is observed in G-CIMP high tumors, in conjunction with 1p/19q deletion, has been shown to sensitize cells to TMZ treatment, leading to significantly improved survival.

Further sub-classification and refinement of subtypes has also required an epigenetic approach. In gliomas, the mutational status of IDH is an important marker, and interestingly, gliomas with mutated IDH also have a particular cytosine-phosphate-guanine (CpG) island methylator phenotype (G-CIMP). The G-CIMP of DNA methylation seems to identify a distinct subgroup of glioma, with G-CIMP “high” subgroup of tumors in younger patients at diagnosis that having better overall prognosis. The G-CIMP “high” phenotype is also more commonly observed in lower-grade gliomas than GBM and tends to have the PN molecular subtype (21, 26). Furthermore, in patients treated with temozolomide (TMZ), those that had recurrences and had lost methylation of the O(6)-methylguanine-DNA methyl transferase (MGMT) promoter, had increased genetic mutations compared to untreated patients, indicating that this methylation phenotype could contribute to the chemotherapeutic resistance of the tumor (21, 26). However, MGMT methylation status is also predictive of treatment response in IDH wild-type GBM patients (36) and abnormal methylation of MGMT has increased prognosis in some GBM patients after TMZ treatment (37) (Figure 1). Recently, small non-coding RNA molecules (ncRNAs or miRNAs) have been suggested to be involved in a number of cancers. Five miRNAs were found to be involved in MGMT alterations and tumor suppressor functions of TP53 (miR-21, miR-125b, miR-34a, miR-181d, and miR-648) in GBM progression (38). In particular, miR-21 and miR-181d were associated with GBM tumorigenesis (39–42), as have a number of other miRNAs, miR-144 and miR-29a (43–45). These miRNAs may prove to be important biomarkers for GBM, but their specificity needs to be further validated.

IDH mutation has been linked with chromosomal abnormalities and prognosis in low-grade gliomas. Correlations have been observed in 3 subtypes: IDH mutant with 1p/19q co-deletion correlating to increase survival (46, 47), whilst IDH mutant without 1p/19q co-deletion and IDH wild-type was correlated with poor prognosis that is similar to GBM (16). Furthermore, patients with oligodendroglioma (which often contain the 1p/19q deletion) tended to respond better to chemo- and radiotherapy, with an enhanced prognosis overall (14, 48). EGFR-TACC fusion via a chromosomal translocation has been described in a small number of GBM patients, but its clinical significance is unclear (35), but may have strong sensitivity to some tyrosine kinase inhibitors (49).

Further studies have identified known oncogenic pathways in GBM such as RB, p53, RTK/RAS/P13K (16); a putative attempt at linking GBM molecular subtypes to cell types of the central nervous system (CNS) has also been suggested based on gene expression signature: PN subtype—oligondendrocytic, CL subtype-astrocytic and MSC subtype–astrocytic (cultured cells) (22, 50). This remains to be fully substantiated. However, the MSC subtype generally is the most heterogeneous, showing its complexity compared to other non-MSC tumors (22). A few studies have also reported a switch between molecular subtypes in recurrent tumors that may be driven by the accumulation of new genetic mutations (23, 51, 52). It has been suggested that recurrent tumors may acquire extra mutations and evolve along two distinct molecular pathways governed by p53 mutation (Type 1 GBM) or EGFR amplification (Type 2 GBM) (51). Although the MSC subtype is the most common subtype in GBM, the shift from PN to MSC has not been clearly shown to occur (15).

Comparative studies between initial and recurrent GBM have been conducted using specific known markers and genome-wide analysis to further understand tumorigenesis and progression. Immunohistochemistry has been used to study proteins thought to be involved in DNA repair and tumor growth such as MutL homolog 1 (MLH1), MutS homolog2 (MSH2), and tumor suppressor p53 (53). These were found to be expressed significantly lower in recurrent GBM. Furthermore, reduction of MLH1 and post-meiotic segregation increased 2 (PMS2) proteins conferred TMZ resistance and is associated with recurrent TMZ (54). Genomic, transcriptomic and epigenetic approaches have been utilized in a number of longitudinal studies using whole epigenome sequencing (WES), targeted genome sequencing (TES), loss of heterozygosity (LOS), quantitative PCR, RNA-Seq, transcriptome profiling and whole genome sequencing (WGS). These studies have identified numerous additional pathways, biomarkers and deciphered the mutational behavior of the tumor with and without treatment. Genetic differences in tumor evolution were observed in primary and recurrent tumors, sharing relatively few initial mutations (55). Subtype switching was also found to be common (66%) in primary GBM and may be a result of accumulation of additional mutations in highly expressed genes (56). A new mutation in latent TGF-β-binding protein 4 (LTBP4) gene was found in 10% of recurrent GBM, whilst the TGF-β pathway was also found to be involved in tumor pathogenesis (56). Primary GBM tumors without p53 and EGFR mutations gain novel EGFR amplification during recurrence and can follow two distinct pathways, depending on the genetic type of the original tumor (51). In another study, using WES, considerable tumor heterogeneity, mediated by EGRF overexpression, was observed in GBM, as well as a deletion on chromosome 10, losing phosphatase and tensin homolog (PTEN) and cyclin-dependant kinase inhibitor 2A (CDKN2A) genes (57). A further study analyzed the evolution of mutations in GBM by using paired samples and found that 67.9% were clonal in nature, whilst 29.8% were sub-clonal (55). Of these, 90% of p53 and PIK3CA/PIK3R1 mutations were also clonal, suggesting that the nature of p53 mutations in GBM has implications for tumorigenesis (55). TMZ treatment also influences the nature and rate of mutations in recurrent GBM tumors (58). Transcriptomic profiling revealed that a macrophage/microglia-rich tumor microenvironment is key for the development of the MSC molecular subtype, which is further facilitated by NF1 depletion (15) (Figure 1).

Epigenomic analysis has offered important insights into molecular mechanisms, such as methylation, underpinning clinical phenotypes. Promoter methylation of the DNA-repair gene MGMT results in gene silencing which was associated with significantly better prognosis in patients treated with TMZ, than those that did not have a methylated MGMT promoter (59). In this study, 45% of 206 GBM cases were found to have MGMT promoter methylation (59). In a recent study, a comprehensive DNA methylation analysis of 200 tumors from 77 GBM patients identified biomarkers which, at the time of diagnosis, were found to be predictive of GBM recurrence and prognosis. Patients in the G-CIMP “high” subgroup, with IDH mutation and intact 1p19q were found to have a good clinical outcome upon recurrence compared to patients with altered and lowered methylation (G-CIMP “low”), at the time of diagnosis, with the latter having an increased risk of recurrence and significantly poorer clinical outcome (27). Another important recent study conducted a detailed survey of DNA methylation in GBM tumors using the reduced representation bisulfite sequencing (RRBS) technique and RNA-Seq, and made significantly findings in dissecting out tumor heterogeneity based on DNA methylation profile (60). Transcriptional subtypes of tumor were identified as well as DNA methylation profiles, predictive of immune cell infiltration, necrosis and tumor cell morphology. Furthermore, de-methylation of Wnt signaling promoters upon recurrence and progression was also associated with worse clinical outcome (60).

These promising studies showing genomic variations, transcriptional profiles, molecular abnormalities of G-CIMP and other global DNA methylation profiles, along with the changes in the local tumor microenvironment, will lead to a greater understanding of the complex tumor-immune heterogeneity, and enable interventions to prevent GBM tumorigenesis and progression in the future (Figure 1). One such key player is the complement system, the most potent and versatile humoral innate immune system.

## Complement System and GBM

**Figure 2:**
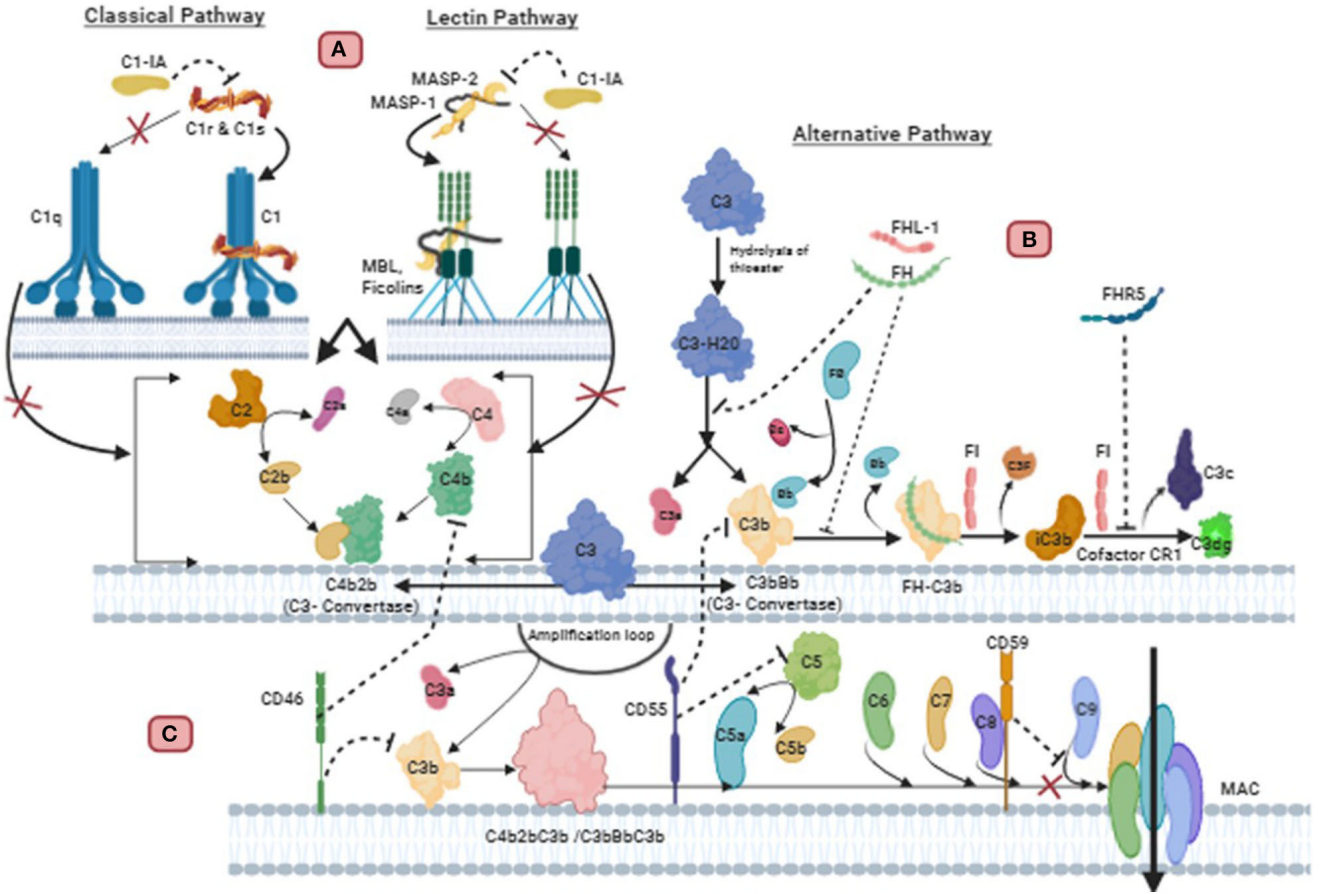
Regulation of complement pathways in Glioblastoma: **(A)** C1 inactivator (C1-IA), also called C1 inhibitor (C1-Inh), binds covalently to the active site of C1r and C1s, blocking their function. It also dissociates C1r_2_C1s_2_ from C1, releasing C1q. This inactivation subsequently prevents the cleavage of C4 and C2 mediated classical pathway. C1-IA can also inhibit the function of MASP-1 and MASP-2 and prevent cleavage of C4 and C2 of the lectin pathway. **(B)** Endogenous or GBM synthesized Factor H (FH) and FH-like protein 1 (FHL-1) can successfully bind to GBM cell membrane. FH is a decay accelerating factor for C3 convertase. This plasma alternative pathway regulator FH binds with C3b in the convertase, displacing Factor Bb to inactivate the convertase. This FH-C3b also acts as a cofactor for cleavage of C3b by Factor I (FI) to yield the inactive product iC3b. CR1 allows FI to perform the second cleavage generating C3c and C3dg. Complement factor H related protein 5 (FHR5) secreted from GBM also exhibits functional activity similar to factor H. FHR5 functions as a co-factor for factor I mediated cleavage of C3b, and decay acceleration of C3 convertase, thus inhibiting complement mediated lysis. **(C)** The membrane bound regulators such as CD59, CD55, and CD46 are also important for resisting complement attack on GBM cells. CD59 binds to C5b-8 complex and blocks the sites for C9 attachment, thus, preventing polymerization of C9 and inhibition of MAC formation. CD55 inhibits the formation and accelerates the decay of C3 and C5 convertase of alternative and classical pathway. CD46 causes inactivation of C3b and C4b deposited on the membrane.

The complement system is one of the first lines of defense of innate immunity in the brain and is comprised of more than 30 different glycoproteins which are soluble proteins, cell associated regulators or receptors (61). Complement can be activated by pathogens and altered-self cells or indirectly by pathogen-bound antibodies. Activation of complement opsonises target pathogens or altered-self cells for phagocytic uptake, inducing an inflammatory response and enabling cell lysis. Complement is activated through 3 different pathways which are the Alternative, Classical and Lectin pathways (Figure 2) (62, 63). The alternative pathway is auto-activated by a process termed ‘tick-over', where C3 (the most abundant complement protein) is spontaneously hydrolyzed, designated C3(H_2_O). Complement protein Factor B associates with C3(H_2_O) and in-turn is cleaved by Factor D generating Ba and Bb. The larger cleaved product Bb remains associated and forms the protease complex C3(H_2_O)Bb which cleaves additional C3 to form the cleaved products C3a and C3b. The cleaved anaphylatoxin C3a can elicit inflammation whereas C3b can bind to and opsonize pathogens and also bind to C3 convertase (C3bBb) to form C5 convertase (C3bBbC3b). An amplification loop can also be initiated when C3b generated from the Classical and Lectin pathway bind with Factor B from the alternative pathway allowing Factor D to cleave it similarly to “tick-over” (63, 64). The activation of the Classical pathway is through the binding of C1q directly to pathogens, altered-self cells or to antibody antigen complexes. This triggers the C1r to activate C1s which cleaves C4 and C2 to generate C4a anaphylatoxin, C4b opsonin, C2a and C2b. C4b and C2b bind to form C3 convertase (C4b2b) (65). Similarly, in the Lectin pathway both C4 and C2 are also cleaved producing the same products that generate C3 convertase (C4b2b). The lectin pathway is activated by mannose binding lectin (MBL) binding to oligosaccharides on pathogens. The associated enzyme mannan-binding lectin serine protease (MASP) 2 are responsible for the cleavage of C4 and C2 (66, 67). All 3 pathways converge at C3 convertase enabling the cleavage of the central complement component C3 to form C3a and C3b. The opsonin C3b binds to C3 convertase and generate C5 convertase (C3bBbC3b) (C4b2Bc3b), which enables the cleavage of C5 to form anaphylatoxin C5a, and opsonin C5b. C5b binds to the pathogen and also to C6, C7, C8, and C9, to produce a membrane attack complex (MAC) which generates pores through the pathogen's cell membrane, leading its destruction by osmotic cell lysis (61).

The complement system plays an important role in defense against pathogens, angiogenesis, neuroinflammation and neurodegeneration, as well as regulation of adaptive immunity. Apart from these functions, complement system also has a key role to play in cancer immunotherapy, cytotoxicity and tumorigenesis (68). Over the years, studies have shown that GBM is resistant to complement-mediated killing and this is facilitated by membrane-bound and soluble complement inhibitors. These regulators include Factor H (FH), FH-like protein 1 (FHL-1), C1 inactivator (C1-IA; also called C1-inhibitor:C1-inh), protectin (CD59), membrane co-factor protein (MCP; CD46) and decay accelerating factor (DAF; CD55) (69–71). FH is an important soluble regulator of the Alternative pathway, as it competes with factor B for C3b binding, to prevent the formation of C3 convertases and thus accelerates the decay of C3 convertase (C3bBb) to disassemble the enzyme (Figure 2). FH also acts as a co-factor for factor I to inactivate C3b by cleaving the α-C3b chain into 2 fragments (72, 73). FH is composed of 20 complement control proteins (CCPs) of which CCP 1–4 facilitate the functional activity of FH. FHL-1 represents the truncated form of FH as its 7 CCPs are identical to the N-terminal of FH, and therefore elicit the same inhibitory ability (73, 74). In the presence of glycosaminoglycans and sialic acid, which are present on self-cells, the affinity of FH increases for surface bound C3b via the 3 binding sites at CCPs 1–4, 7–15, and 19–20. The polyanions are only present on self-cells, thus enabling FH to differentiate between self and non-self-cells (72, 75).

### Complement Regulators

Complement regulatory proteins are important in protecting healthy self-cells from complement attack by exerting tight regulatory functions. Regulation is required at all major checkpoints of complement activation and amplification to prevent a deleterious effect on self-cells from an over-reactive complement system. Healthy cells express soluble regulators such as FH and membrane bound regulators including CD59, CD55, and CD46 (Table 2), which all use different mechanisms to provide protection (91, 92). Soluble regulators inactivate complement as they are attracted to self-structure over foreign surfaces (93, 94). However, soluble and membrane-bound complement regulators can act as double-edged swords by overregulating the complement system to the point it is unable to eliminate tumor cells. Studies suggests that the expression of complement regulators by tumors including GBM allows these cells to proliferate unchecked. This highlights the significance that complement regulators play in the tumor cells' avoidance of complement attack. As knowledge of the relationship between complement regulatory proteins and tumors evolves, it is possible that their therapeutic blockade can have an important role in tumor treatment (70, 71).

**Table 2:** Immune system components associated with Glioblastoma multiforme (GBM) microenvironment.

**Immune system component**	**Source**	**Effect on GBM microenvironment**	**References**
**Cytokine**			
IL-10	TAM	Enhances Immunosuppression, promotes tumorigenesis, decreases expression of MHC class II on monocytes, promotes Tregs, inhibits expression of TNF-α and IFN-γ, suppresses anti-tumor effect of immune cells	(76–78)
TGF-β	TAM and GSC [TGFB2]	Suppresses anti-tumor immune response, promotes tumorigenesis, blocks NK cells activity, Inhibits T-cells, promotes Tregs, downregulates IL-2, Inhibits NKG2D on CD8^+^ T-cells, upregulates CD133^+^	(79–83)
IL-6	TAM	Suppresses immune effector cells	(84, 85)
CSF-1	TAM	Enhances immunosuppression	(86–88)
**Complement system**			
FH	GBM cells	Enhances immunosuppression, inactivates C3b, inhibits activation of the complement alternative pathway	(70)
C1-IA	GBM cells	Enhances immunosuppression, prevents activation of the complement classical pathway	(69)
CD59	GBM cells	Enhances immunosuppression, inhibits the formation of MAC, prevents activation of the complement pathway	(70)
CFHR5	GBM cells	Inhibits complement-mediated lysis and decay acceleration of C3 convertase	(89)
**TAM**			
TAM	Microglia and macrophage/ monocyte	Polarises toward M2 phenotype, enhances immunosuppression, promotes tumor invasion, secretes anti-tumor cytokines, expresses FasL which act as an immunosuppressant, expresses MMPs which promote tumor invasion, promotes proliferation of growth factors	(86, 90)

*IL, interleukin; TGF, transforming growth factor; CSF, colony stimulating factor; FH, factor H; C1-1A, complement 1-inactivator A; CFHR5, complement factor H related protein 5; TAM, tumor-associated macrophage*.

### Factor H

Factor H is secreted by GBM cell lines such as H2, U138, U118, and U87 (95). In another study by Junnikkala et al., expression of RNA and protein production of FHL-1 in the malignant cells was found to exceed that of FH, in contrast to normal serum where the concentration of FH is greater than FHL-1 (70) (Table 2). It appears that endogenously synthesized and fluid phase FH and FHL-1 from plasma can successfully bind to the GBM cell membrane, efficiently regulating complement activation and promoting the cleavage of membrane deposited C3b into its inactive form iC3b. Ultimately, this mechanism prevents activation of the late stages of complement activity, to elicit cell lysis via MAC formation because there is reduced C5b-9 deposition. The inhibitory effect of secreted FH and FHL-1 can be overcome through neutralization of FH and FHL-1 with antibodies that target the C3b binding site and by the removal of sialic acid to sensitize GBM cells to complement lysis. FH and FHL-1 play a crucial role in GBM tumorigenesis by enabling the acquisition of GBM cells' exceptional resistance to complement mediated killing (70). In a more recent study on primary tumor cells derived from 3 GBM patients, secretion of complement Factor H related protein 5 (FHR5) was also reported (89). It was found that the cells secreted FHR5, but not FH, and that FHR5 inhibited complement-mediated lysis and decayed acceleration of C3 convertase (89).

### Complement 1 Inactivator A

GBM resistance to complement-mediated lysis can be acquired by the production of Complement 1 inactivator (C1-IA) or C1 inhibitor (C1-inh) (Table 2). C1-IA, a serine protease, is able to regulate classical pathway activation by irreversibly binding to C1r and C1s proteases, which along with C1q, form the multiprotein complex C1, which is the first component in the initiation of the classical pathway (96, 97). The ability of C1-Inh to bind to C1r and C1s protease subsequently prevents C1r autoactivation and C1s activation, which in turn, prevents the cleavage of C4 and C2. This ultimately stops the formation of the Classical pathway's C3 convertase (C4b2a) (98). Gene expression and mRNA analysis in human GBM tissues showed an upregulation of C1-inh (69). Inhibition of C1-inh in rats with GBM, using appropriate antibodies, was found to increase survival but also led to decreased levels of cytokines IL-1β and GM-CSF, which are associated with an immunosuppressive tumor microenvironment (69, 99).

### Membrane-Bound Complement Regulators

The ability of GBM cells to avoid complement attack is not only determined by soluble inhibitors but also by membrane bound regulators such as CD59, CD55, and CD46 (70, 71) (Table 2). CD59 is a major protective element against complement mediated lysis. It binds to C5b-8 complex and blocks the sites to which C9 can attach, thus, preventing the insertion and polymerization of C9. As a result, the final step of MAC assembly on the cell membrane is prevented (100). CD55 is an anchored membrane regulator that inhibits the formation and accelerates the decay of C3 and C5 convertase of the alternative and classical pathway to prevent complement activation (101). The complement cascade is also regulated by CD46, which serves as a co-factor of factor I inactivation of C3b and C4b, deposited on the membrane (102).

In a study by Maenpaa et al., it was shown that CD59 was expressed in 14 human glioma tissues as well as 7 glioma cell lines (71). In normal astrocytes, the expression of CD59 is weak as the need to protect these cells from complement is reduced due to the blood-brain barrier, which restricts entry of many pathogens into the brain (71). Successful binding of CD59 to C5b-8 complex inhibits the formation of MAC at the point of insertion of C9 into GBM cell membrane, thus protecting the cell from complement mediated killing (70). The inhibition of CD59 by neutralizing antibodies enables the cells to overcome the resistance of GBM to complement mediated cytolysis (70). In the same study, CD55 and CD46 were also shown to be moderately expressed in GBM cell lines, and neutralizing them with respective antibodies showed moderate complement-mediated cytolysis, although CD59 was considered to be the most important complement regulator on GBM cells (70).

### Role of Microglia and Macrophages in GBM

The CNS has historically been considered an immune privileged site. This is primarily because it lacks a traditional lymphatic system, containing only a few antigen presenting cells which would mount an extremely weak immune response (103). Considering recent data, the characteristics of immune privilege have been redefined and are no longer considered absolute (103). The concept of immune privilege had stemmed from the ability of antigens within the brain to avoid systemic immunological recognition (104). It is now evident that immune privilege is specific to brain parenchyma which is imperative for damage limitation during inflammation. The brain parenchyma is an extremely sensitive part of the organ with poor regenerative capacity and is protected by the blood brain barrier, a semi-permeable membrane consisting of endothelial cells that separate the blood from the cerebro-spinal fluid (104).

The CNS is able to coordinate a robust immune response involving both the innate and adaptive immune systems (105). During inflammation, immune cells are able to migrate to perivascular spaces following chemotaxis (106). Studies have shown that antigens can enter the cervical lymph nodes by passing through the Virchow Robin Perivascular Space within the walls of the cerebral arteries (107). It is also possible for immunoglobulins to cross the blood-brain barrier via carrier mediated transporters by attaching to FcRn receptor (108). Antigen presentation occurs as dendritic cells (DCs) can travel outside of the brain and present antigens to T-cells located in the cervical lymph nodes (109). However, inflammation and disease in the CNS can compromise the integrity of the blood-brain barrier, thereby enabling circulating immune cells to migrate past it and infiltrate the parenchyma (110).

Microglia are the resident macrophage of the CNS comprising 5–20% of the total glial cell population. In the brain, microglia are involved in immune surveillance and are a crucial component of the first line of defense (111). Originally discovered over a century ago by Pio Del Rio Hortega, it is now clear that resident microglia originate from haematopoietic precursor cells of immature yolk sac during early embryogenesis (112). Microglia are usually found in a “resting” state; microglia having branched extensions or processes actively patrol and perform surveillance of local areas. Following inflammatory stimuli, inflammatory stimuli, circulating microglia change into “amoeboid” shape, and additional recruitment of macrophage from infiltrating circulating monocytes takes place (113, 114). Apart from surveillance, microglia actively contribute to brain development and CNS homeostasis by apoptotic cell removal, maintenance and pruning of synapses, and regulation of neuronal activity (114, 115). In GBM, a second group of macrophages derived from peripheral bone marrow, are present (116). In the brain, macrophages are restricted to the perivascular, choroid and meningeal locations. However, disruption to the blood-brain barrier by disease or inflammation allows macrophage to gain entry to the parenchyma (117). These mononuclear cells are difficult to differentiate from microglia as they intermingle in GBM (118).

**Figure 3:**
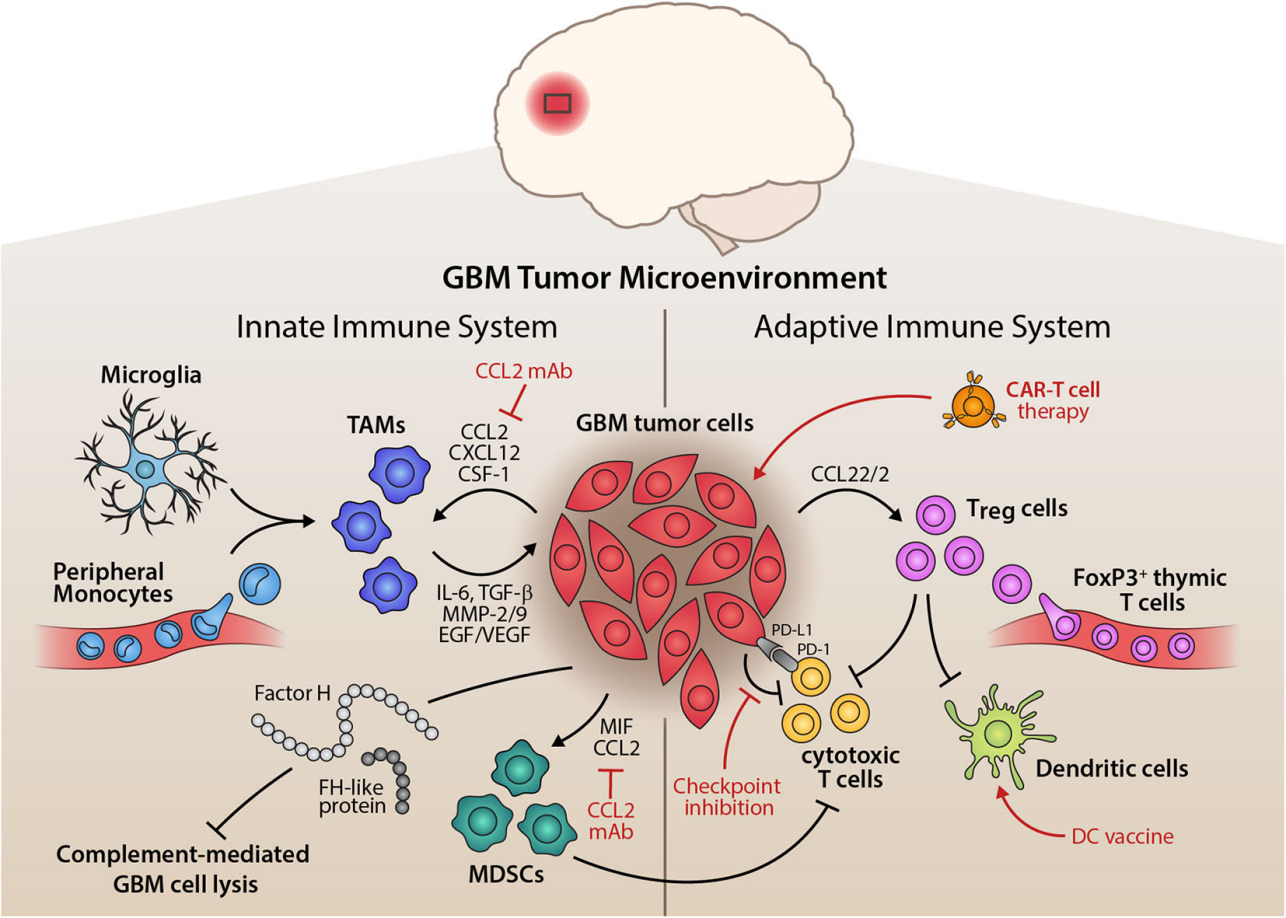
Inflammatory Tumor Microenvironment of GBM and its Therapeutic Implications. Illustration of the interplay of innate and adaptive immune components within the glioma microenvironment. On the side of the innate immune system, tumor-associated macrophages (TAMs), mainly comprised of microglia and peripheral monocytes, are attracted by tumor cells, which release pro-inflammatory cytokines, matrix remodelers, and growth factors to aid tumorigenesis. Myeloid-derived suppressor cells (MDSCs) are also recruited by the tumor and potently suppress anti-tumor immunity. Alternative pathway molecules factor H (FH) and FH-like protein 1 of the complement system enhance immunosuppression and prevent complement-mediated lysis of the tumor cells. The adaptive immune system, on the other hand, is largely suppressed in its function through the recruitment of regulatory T cells (T_reg_). These inhibit the action of cytotoxic T cells and dendritic cells, disturbing a competent anti-tumor immune response. Tumor cells also exert direct suppression of adaptive immunity through immune checkpoint expression, e.g., PD-L1 or CTLA-4. Therapeutically, this tumor-immune crosstalk can be targeted by inhibiting chemoattractants of pro-tumor immune cells, such as anti-CCL2 monoclonal antibody, by immune checkpoint inhibition, dendritic cell vaccination approaches or adoptive transfer of chimeric antigen receptor (CAR) T cells that target the glioma cells (red indicators).

Traditional approaches to distinguish macrophage and microglia involved use of CD45 antibody as microglia are defined as CD45^low^, whereas macrophages are defined as CD45^high^ (118, 119). Despite this, it is still unclear as to whether microglia or macrophage make up most of the mononuclear density in GBM. Parney et al. suggested that gliomas contained more recruited macrophages than resident microglia (120). However, Muller et al. challenged this concept as they demonstrated resident microglia were the main source of mononuclear cells in gliomas and that the microglia present had increased their expression of CD45 (121). Together, microglia and macrophages in GBM are generally referred to as tumor-associated macrophages (TAM) (Figure 3) (122).

It has also been reported that in the MES subtype, deficiency of NF1 leads to increased infiltration of TAM (15). This may explain why GBM subtype-specific cell autonomous functions drive tumor aggressiveness and therapy resistance and have poorer prognosis. Furthermore, this study also highlighted that the tumor microenvironment in recurrent GBM showed the presence of more resident microglia/macrophages as compared to peripherally-derived monocytes, indicating that treatment (such as radiotherapy) may have an impact on monocytes, and thus in recurrent GBM; more efforts need to be made to address resident cells in the brain. This elegant study also showed increased CD8^+^ T cells in TMZ-induced hypermutated recurrent GBM (15).

Microglial cells have been known to enhance infiltration leading to increased invasiveness of the tumor. A murine microglial cell study on mouse glioma cells found that tumor cell migration occurred sooner and was higher when compared to tumor cells without microglia (123). Another study using murine brain slices found that microglia stimulated the extracellular matrix metalloprotease (MMP)-2, which led to increased invasiveness of the tumor (124). Pro-inflammatory cytokines such as IL-1β, IL-6 and TNF-α, secreted by microglia, have been shown to increase tumor invasiveness *in vitro* (125). By specifically targeting microglia, using propentofylline which blocks secretion of IL-1β, IL-6 and TNF-α, tumor growth was found to regress (126).

GBM cells secrete a range of chemo-attractants such as CCL2, CXCL12, and SDF-1, which actively recruit microglia and macrophages (127, 128). Various CC and CXC chemokines are secreted including CCL2, CXCL12, and their receptors (129, 130). CCL2 is one of the most important CC chemokines commonly expressed by GBM as it plays a key role in regulating the penetrative migration of TAM to the GBM microenvironment (131). It was the first TAM chemo-attractant identified in GBM; the level of CCL2 expression is associated with glioma grade (132). CCL2 is highly expressed in GBM at mRNA and protein levels, thus contributing to a high influx of TAM (133). Inhibiting CCL2 activity in mice studies (GL261 glioma and xenograft of human U87 models) with relevant antibodies has been shown to reduce infiltration and ultimately prolong survival (134). The receptor for CCL2 is CCR2 which are also present on microglia (135). In addition, microglia from the GBM tumor microenvironment have the capacity to secrete CCL2, thereby stimulating more microglia recruitment to the tumor (130).

CXCL12, also known as stromal derived factor 1 (SDF-1), a chemokine, promotes TAM recruitment in high-grade gliomas. A murine high-grade model, ALTS1C1, demonstrated the chemo-attractant ability of SDF-1 for microglia and macrophages. High expression of SDF-1 promoted the accumulation of TAM to areas of hypoxia in brain and tumor invasion (136). GBM cells also express colony stimulating factor-1 (CSF-1) which functions as TAM chemo-attractant (86, 87). CSF-1 is overexpressed in GBM, thus contributing to the high influx of microglia/macrophages, promoting tumor invasion (86, 87). High glucose has been shown to increase proliferation and inhibit apoptosis in a study on human GBM U87 cell line, by upregulation of vascular endothelial growth factor (VEGF) and is mediated by increased expression of chemotactic receptors including EGFR (137). A recent murine study showed that osteopontin is an important chemokine that attracts TAM to the GBM site, via integrin α_v_β_5_ (138). Further, α_v_β_5_ deficiency was found to lead to a direct CD8^+^ T cell cytotoxic effect at the tumor site (138).

Majority of newly recruited TAMs acquire an alternatively activated M2 phenotype under the direct influence of tumor cells to produce a pro-tumor microenvironment. M2 polarized TAMs produce mediators that contribute to the immunosuppressive microenvironment established by the tumor cells (139). TAMs are known to secrete anti-inflammatory cytokines such as IL-6, IL-10 and TGF-β, thereby enhancing immunosuppression in tumor microenvironment, leading to promotion of GBM cell growth and angiogenesis (84). Studies have shown that these anti-inflammatory cytokines supress M1 phenotypes as TGF-β inhibits pro-inflammatory cytokine expression and microglia proliferation whilst IL-10 polarizes microglia to a M2 phenotype (88). TAMs are also known to express Fas ligand (FasL) which acts as an immunosuppressant in GBM, as it contributes to the reduced presence of tumor infiltrating leukocytes (90).

The pro-tumor microenvironment of GBM is supported by the expression of MMPs by TAM, including MMP-2 and MMP-9, which are involved in tumor growth by having an impact on angiogenesis, apoptosis and cell proliferation (140). Subsequent inhibition of MMPs derived from TAM have shown a reduction in tumor growth and angiogenesis (141, 142). A study has shown that membrane type 1 (MT1) MMP is enhanced in TAM, which in turn, activates MMP-2 in GBM, via microglial cells, thus increasing tumor invasion (143). TGF-β1 derived from microglia in GBM plays an important role in TAM-mediated promotion of tumorigenesis (79). It has been shown that TGF-β1, released by TAM, induces Epithelial-to-Mesenchymal Transition (EMT) and enhanced invasion of CD133^+^ Glioma stem cells (GSCs) which led to a pro-tumorigenic environment (80). Moreover, TAMs also contribute to tumorigenesis in GBM by providing proliferation promoting factors such as EGF and VEGF (86).

IL-10 from TAM in GBM have the ability to promote tumor growth i*n vitro* via JAK2/STAT3 pathway (76). Activation of Signal Transducer and Activator of Transcription 3 (STAT3) co-ordinates the expression of immunosuppressive molecules by decreasing expression of major histocompatibility complex (MHC) class II and co-stimulatory molecule, CD40 (77). An activation loop is formed as the stimulation of STAT3 by IL-10 enables activation of this transcription factor in nearby immune cells (77). These cells include macrophage, natural killer (NK) cells and DCs. As a result, the anti-tumor activity of these immune cells is supressed (78). IL-10 derived by TAM also supresses MHC class II expression on monocytes and down-regulates the production of IFN-γ and TNF-α in GBM, thus preventing anti-tumor activity (144). The overall effect of IL-10 secreted by TAM on GBM is immunosuppression which ultimately promotes a pro-tumor milieu (145).

DCs are antigen-presenting cells, involved in surveillance against pathogens and tumorigenic cells, and present these to T cells, thereby serving as an important link between innate and adaptive immunity. This is utilized in anti-tumor therapies, to help induce a cytotoxic response against the tumor cells. In GBM, DCs are considered to present tumor cell peptides, leading to cytotoxic T cells response, and secretion of pro-inflammatory cytokines. Pre-clinical studies on murine glioma models have found DCs to be effective in inducing an effective tumor-response and increasing survival (146, 147). Phase I clinical trials have shown DC vaccination therapy to be safe and to elicit cytotoxic T cell responses (148, 149). Early results from a subsequent Phase III clinical trial involving an autologous tumor-lysate pulsed DC vaccine was shown to be feasible and safe and may extend survival in GMB (150).

Microglia in GBM are a major source of TGF-β, which plays a key role in contributing to the immunosuppressive GBM microenvironment (135). TGF-β enhances immunosuppression in GBM through a range of mechanisms including blocking T-cell activation and proliferation, inhibiting the activation of NK cells, down regulating IL-2 production, and promoting T_regs_ (81). Blocking T cell activation can be achieved by the ability of TGF-β2 to supress HLA-DR antigen expression which is essential for tumor associated antigen presentation to CD4^+^ T-cells (82). TGF-β is also capable of facilitating immune escape by inhibiting NKG2D (an activating receptor responsible for host-response to pathogen and tumor cells) on CD8^+^ T cells and NK cells ultimately rendering the cells less effective at cytotoxic destruction of GBM (83). Strategies which inhibit TGF-β expression can restore anti-tumor immunity in GBM. Transient silencing of TGF-β, using siRNA, has been shown to prevent NKG2D expression and increase GBM susceptibility to destruction by immune cells (151). Murine glioma models also showed that blocking TGF-β1 receptor increased the number of long-term survivors by 33%, as opposed to the 6% observed in the control group. The level of CD8^+^ T cells were also increased, demonstrating a reversal of the immunosuppressive effect when TGF-β1 is inhibited (152).

NK cells are known for its anti-viral and anti-tumor response, and secrete cytokines such as IFN-γ and TNF-α. Pre-clinical models of GBM have shown NK cells to be effective in HLA class I-mediated tumor lysis (153); IL-2 activated NK cells' ability to kill GBM cells (154), and NK cells' effectiveness in preventing metastasis in the GBM xenograft mouse model have been reported (155).

## Adaptive Immunity and T_reg_ Cells

T_reg_ cells play a major role in mediating immune suppression of anti-tumor immune cells. In non-tumorigenic environments, T_regs_ usually are involved in preventing autoimmunity (156). T_regs_ are a sub-population of CD4^+^ T-cells and can be categorized into two groups based on their developmental origin. Thymus derived T_regs_ develop after antigen presentation by thymic epithelial cells and are characterized by high level expression of the transcription factor Forkhead Fox P3 (FoxP3) (157). By contrast, peripherally induced T_regs_ differentiate in the periphery upon antigen presentation and recognition by naive conventional CD4^+^ T-cells. IL-10 and TGF-β signaling are key contributors in supporting the induction of peripherally induced T_regs_ which have negligible FoxP3 expression (158). Studies have shown that there is a high influx of T_regs_ predominately of thymic origin, accounting for 25% of tumor infiltrating lymphocytes (159, 160). The abundance of T_regs_ is associated with poor prognosis, as they shift the tumor cytokine milieu toward immunosuppression (161). This enhanced immunosuppression is achieved by T_regs_ ability to restrict the function of infiltrating T cells by preventing production of IL-12 (162). The high influx of T_regs_ in GBM is likely due to CCL22 and CCL2 secreted by GBM, as they bind to CCR4 commonly expressed by T_regs_ (163, 164).

### Immune Checkpoint

Immune checkpoints are co-stimulatory and co-inhibitory pathways that restrict the function of the immune system. These regulatory pathways supress T-cell activation and proliferation, ensuring that immune responses are limited to maintaining self-tolerance which prevents the immune system attacking self-cells (165). An immune checkpoint involved in GBM immune evasion is programmed cell death protein 1 ligand (PD-L1), which is a transmembrane glycoprotein of the B7 family co-stimulatory molecules (166). PD-L1 is not usually expressed in the CNS, therefore, its presence in this location is associated with a pathological or tumorigenic environment (167). PD-L1 is activated by binding to the receptor programmed cell death protein 1 (PD-1) to exert its inhibitory effect (168). In GBM, activation of PD-L1 suppresses the proliferation and function of tumor resident cytotoxic T cells, which would otherwise destroy the tumor cells. PD-L1 can also enhance T_reg_ activity which will promote a pro-tumorigenic microenvironment (168) (Figure 3).

Various immune cells express PD-L1 in GBM, such as CD4^+^ and CD8^+^ T cells (169). TAM express PD-L1 on their surfaces, whilst promoting PD-L1 expression on GBM cells (166). Genetic alterations have also been shown to contribute to PD-L1 expression as the loss of PTEN tumor suppressor gene enhances the expression of PD-L1 on glioma cells (170). The expression pattern of PD-L1 is positively correlated with glioma grade and is also associated with poor survival of GBM patients (169). A study in mouse glioma cell-line has shown that inhibiting PD-L1 with antibodies on glioma cells in combination with radiotherapy has clear survival benefits (171). PD-L1 expression was found to be dependent on IL-6; inhibition of IL-6 signaling diminished expression of PD-L1, leading to increased survival and reduced tumor growth in orthotopic murine glioma model (85).

Cytotoxic T Lymphocyte Antigen 4 (CTLA-4) is another immune checkpoint molecule which plays a role in GBM immune evasion, as it modulates the early stages of T lymphocyte activation. CTLA-4 is expressed on activated T-cell and T_reg_ in a tumor microenvironment (172). Targeting CTLA-4 in glioma models with anti CTLA-4 antibodies proved useful in reversing immune evasion. This study showed an increase in long term survival, increased resistance to T_reg_ mediated suppression and enhanced proliferation of CD4^+^CD25^−^ T-cells (172).

Despite several biological and clinical approaches, including the 2018 Nobel Prize for immune checkpoint blockade in cancer immunotherapy, no specific immune therapy treatment for GBM has been successful in phase III or randomized controlled trials due to either lack of positive response, or due to side-effects (173). Some of the clinical trials that did not show significant survival benefit include nivolumab (anti-PD-1) and ipilimubab (anti-CTLA-4) in recurrent GBM (174); nivolumab vs. TMZ and radiation therapy in newly-diagnosed GBM (175); and nivolumab in combination with TMZ and radiation therapy in newly-diagnosed GBM (176).

Other emerging themes in cancer immunotherapy include inhibition of VEGF to reduce angiogenesis and vascular permeability, and cancer vaccine-based therapy such as use of DCs to activate T cells (173). The overall survival and progression-free survival was found to be increased in newly diagnosed GBM patients who received TMZ, GM-CSF, and targeted cytomegalovirus (CMV) with DCs (177). CMV proteins have been found to be expressed in GBM but not in normal brain tissue, and this has been utilized to generate specific T-cell immune response to lyse GBM tumor cells (178). A follow-on randomized trial in GBM patients showed significant progression-free and overall survival in patients who received CMV-specific DC vaccination (179). Another exciting theme involves use of CART-cell therapy (chimeric-antigen receptor T-cell therapy), in which immune receptors are specifically engineered to generate an immune response when they face tumor proteins (180). A study in recurrent GBM patients, targeting a type of EGF, using CART-cell therapy, was found to kick-start an immune response at the site of the glioma including infiltration by T_reg_ cells (181). This preliminary study is the first in humans and involved 10 patients with recurrent GBM. They were treated with a single peripheral dose of autologous T-cells targeted to EGFR variant III, which is found in about 30% of GBM patients and associated with poorer prognosis (182). This particular CART-cell therapy was found to be safe, the infused product reached tumor site in the brain, and also found to assert anti-tumor activity by decreasing EGFR variant III expression (Figure 3).

### Glioma Stem-Like Cells (GSCs)

Cancer stem cell hypothesis relates to presence of cells with stem-cell like properties in the tumor microenvironment (i.e., cells that possess ability to differentiate into various cell lineages or generate new tumor or resistance to treatment) (183). The GBM microenvironment is thought to contain such cells called as GSCs that possess properties of self-renewal, pluripotency or ability to give rise to differentiated cell types, and resistance to multiple drug and radiation therapy. The presence of GSCs in GBM was first discovered by Singh et al., and since then numerous studies on GBM microenvironment have established their role in therapeutic resistance, tumor migration and invasion, capability to metastasise, as well as continued maintenance of stem cell-like state of cells (35, 184).

GSCs are considered to have the ability to escape immune response by down-regulating expression of MHC class I, thereby leading to failure of activation of cytotoxic T cells (185). One of the important mechanisms involves PD-L1 present on extracellular vesicles (lipid membrane-bound vesicles secreted by cells; also called exosomes and microvesicles) secreted by GBM cells, which block T-cell receptor by anti-CD3, thereby reducing activation and proliferation of CD4^+^ and CD8^+^ T cells (186). GSCs have also been shown to evade immune response by increasing production and infiltration of T_reg_ cells (83), and by increasing levels of TGF-β produced by TAM, which in turn, increase levels of TGF-β, thus, down regulates MHC II and subsequent antigen processing mechanism, causing T-cell anergy (187). GSCs are known to attract TAM *in vitro* via CCL2 and periostin (188) and by secretion of cytokines TGF-β and CSF, which are known to polarize TAM to immunosuppressive mode (88).

### Myeloid-Derived Suppressor Cells (MDSCs) in the GBM Microenvironment

One of the major characteristics of GBM is the abundance of Myeloid-derived suppressor cells (MDSCs) in the tumor microenvironment, which largely determines disease prognosis by immune suppressive functions. MDSCs are the key components of innate immune system which essentially originate from the bone marrow derived cells. Significantly, infiltrations of MDSCs in GBM tumor microenvironment were markedly associated with cytotoxic T cells suppression (189, 190). A recent study showed that MDSCs substantially paralyze CD4^+^ T cell memory functions in GBM patients (191). Moreover, findings in GBM murine models showed that pharmacological targeting of MDSCs by Sunitinib resulted in significantly increased CD3^+^CD4^+^ T cell count in the tumor microenvironment (189, 190). Moreover, MDSCs depletion led to improved animal survival as well as increased T cell activation in the in GBM patients' PBMCs (189, 190). Within GBM, GSCs constitute the major neoplastic compartment, which substantially modulates immune suppressive functions by recruitment of non-neoplastic components such as MDSCs, TAMs, and T_regs_ in the tumor microenvironment (192–195). Previous studies have reported that GSCs produce intrinsic factors such as IL-10, IL-4Rα, and TGF-β to program M2 macrophages and activation of T_reg_ cells for an effective immunosuppressive function (188, 192, 194–196). In solid tumors, cell-intrinsic factors of the neoplastic compartment play a key role in recruiting TAMs and MDSCs for disease progression. For instance, CC chemokine CCL2 (MCP1) is the most abundant chemokine, which significantly correlated with poor prognosis in GBM patients (130, 197). Genetic depletion of CCL2 in the murine model is associated with reduced infiltrations of MDSCs in the GBM microenvironment (198). CCL2 depletion led to a significant recruitment of cytotoxic T cell in the tumor microenvironment, which resulted in glioma growth suppression (198). The immunosuppressive functions of CCL2 is mediated through its binding to CCR2 and CCR4 receptors, which are mainly expressed on T_regs_ and MDSCs in GBM, respectively. Moreover, high expression of CCL2 in the GBM microenvironment leads to infiltration of T_reg_ cells, MDSCs, and TAMS, which subsequently is associated with poor GBM prognosis (130, 163, 198). GSCs produce macrophage migration inhibitory factor (MIF), which recruits MDSCs for immunosuppressive functions and GSC proliferation (195). In addition, TAMs and MDSCs account for up to 50% in the immune compartment of GBM microenvironment; in particular, MDSCs are the main source of TGF-β and PD-L1 (191, 199, 200). Hence, from a clinical viewpoint, targeting the CCL2-CCR axis, MIF, and PD-L1 could potentially offer effective therapies for GBM patients.

Unfortunately, the outcome of recent clinical trials of immunotherapies in GBM did not show any promising results. Therefore, personalized immunotherapy in combination with chemo-radiotherapy strategies for GBM patients are currently under consideration. In line with this, findings from the most recent preclinical study confirmed that combining immuno-radiation therapy exclusively targeting MDSCs and TAMs, did result in improved survival, compared to the monotherapy cohort (194, 201). Collectively, interfering with both cell-intrinsic factors of neoplastic compartments and immunosuppressive components (e.g., MDSCs) of the tumor microenvironment might offer an effective strategy to block GBM progression and overcome resistance to conventional therapies.

## Conclusions

This review highlights the molecular determinants of the complex heterogeneous tumor-immune environment observed in GBM and the mechanisms and interactions of various genetic pathways, transcriptional programming, immune cells and the role of the immune suppressive microenvironment in Glioblastoma. Each aspect of metabolic pathways, innate and adaptive immune responses (including complement system) have a key role to play in the initiation, progression, infiltration, maintenance and suppression of tumor cells, thereby continuing to provide hope for potential effective therapies in future. The multi-dimensional interactions of glioma cells along with immune cells and other metabolic pathways add to the complexity of finding successful treatment avenues. Further research into this interplay of the immune response in GBM, along with the genomic processes underlying this, together with parallel progress in clinical trials, is required to overcome this lethal disease.

## Author's Note

The authors would like to dedicate this article to the loving memory of *George Antoni Tsolaki* who died of Glioblastoma in February 2010.

## Author Contributions

SD and AS wrote the first draft. AT added molecular concepts. SS and UK reviewed and edited. HY and LK drew illustrations and added supplementary information at the revision stage. All authors contributed to the article and approved the submitted version.

## Conflict of Interest

The authors declare that the research was conducted in the absence of any commercial or financial relationships that could be construed as a potential conflict of interest.
